# Impure Air

**Published:** 1891-03

**Authors:** 


### IMPURE AIR.

To exclude the cold on severe nights, bedrooms are kept shut tight, and the occupant breathing the confined air all night renders it impure. To avoid this evil other persons ventilate their rooms more than is necessary and catch cold. A simple test to know if the air of the room is sufficiently pure is the following: Fill a quart bottle and take it into the room the air of which we wish to examine; empty the water and the bottle is immediately filled with the air. Then pour into the bottle a spoonful of clear lime water and shake it.

If the air is sufficiently pure, the lime water will remain clear ; but if there is too much carbonic acid in the air, the lime water will become clouded or milky. A few trials of this kind in different rooms will give the experimenter a good knowledge of the purity or the impurity of the air of the rooms. A convenient way is to fill the bottle with water over night, carry it into the bedroom, empty it out the first thing when you get up in the morning, cork the bottle tight, and try the lime water in broad daylight.

If the lime water remains clear, a single breath of air blown into it through any tube or straw will quickly show the chalk in the water, and indicate how readily the air is vitiated by breathing. It is worth while to try the experiment with lime water in a crowded school room, after being occupied several hours, to see if the scholars are not fed with poisoned air, giving them the headache, and rendering it impossible for them to study ; or on the other hand chilling them with needless cold currents.

